# Dropped Head Syndrome Attenuation by Hybrid Assistive Limb: A Preliminary Study of Three Cases on Cervical Alignment during Walking

**DOI:** 10.3390/medicina56060291

**Published:** 2020-06-12

**Authors:** Hideki Kadone, Kousei Miura, Shigeki Kubota, Tetsuya Abe, Yukiyo Shimizu, Yasushi Hada, Kenji Suzuki, Yoshiyuki Sankai, Masao Koda, Masashi Yamazaki

**Affiliations:** 1 Center for Innovative Medicine and Engineering, University of Tsukuba, Tsukuba 305-8577, Japan; 2 Center for Cybernics Research, University of Tsukuba, Tsukuba 305-8577, Japan; kenji@ieee.org (K.S.); sankai@golem.kz.tsukuba.ac.jp (Y.S.); 3 Department of Orthopaedic Surgery, University of Tsukuba, Tsukuba 305-8577, Japan; au.l1ke.color@gmail.com (K.M.); s-kubota@md.tsukuba.ac.jp (S.K.); Abetetsu@tsukuba-seikei.jp (T.A.); masaokod@gmail.com (M.K.); masashiy@md.tsukuba.ac.jp (M.Y.); 4 Department of Rehabilitation Medicine, University of Tsukuba, Tsukuba 305-8577, Japan; shimiyukig@md.tsukuba.ac.jp (Y.S.); y-hada@md.tsukuba.ac.jp (Y.H.)

**Keywords:** dropped head syndrome 1, hybrid assistive limb (HAL) 2, wearable robot 3, cervical alignment 4, cervical kyphosis 5, walking 6

## Abstract

*Background and Objectives:* Dropped head syndrome (DHS) is characterized by apparent neck extensor muscle weakness and difficulty in extending the neck to raise the head against gravity. DHS affects forward vision and eating behavior, and hence impairs quality of life. However, standardized treatment of DHS has not yet been established. The purpose of this preliminary study is to seek for a possibility of effective non-surgical, conservative treatment for DHS, by applying a robotic treatment. *Materials and Methods*: A wearable exoskeleton type robot suit hybrid assistive limb (HAL) was applied to three patients with DHS. A course of HAL treatment included 10 sessions of gait training using HAL. One session lasted about an hour. Case 1 completed the course twice, the first time in two weeks (one session per day) and second time in 10 months (one session per month). Case 2 and Case 3 completed the course once in 10 weeks (one session per week) and in 6 months (one session per 2.5 weeks), respectively. Immediate and lasting effects of HAL on the reduction of cervical sagittal vertical alignment (SVA) during gait was evaluated using a motion capture system. *Results*: Case 1 showed improvement of cervical alignment during gait after the HAL courses of both different frequencies. Case 2 did not show improvement of cervical alignment during gait. Case 3 showed improvement of cervical kyphosis but not of cervical sagittal alignment during gait. *Conclusions*: The results of the preliminary study suggest that gait training using HAL may be an effective option of conservative treatment for a part of DHS patients. They also suggest that a lack of immediate effects on the cervical alignment and a lack of ability to perform compensatory trunk motion may indicate a non-responding patient. Generalization of the results requires further research with more cases.

## 1. Introduction

Dropped head syndrome (DHS) is characterized by apparent neck extensor muscle weakness and difficulty in extending the neck to raise the head against gravity, causing a resultant chin-on-chest deformity. Although this symptom can be passively corrected [1], it severely affects forward vision, causes neck pain and difficulty eating, and therefore impairs activities of daily living (ADL) and quality of life (QOL) [2]. Etiology of DHS is various, including isolated neck extensor myopathy (INEM), Parkinson’s disease, myasthenia gravis, amyotrophic lateral sclerosis [1]. Treatment of DHS is not yet established [3]. A systematic review showed that surgery is the most successful treatment with positive clinical outcome [2]. On the other hand, there are several reports showing efficacy of rehabilitation therapy for DHS including bracing and neck extensor muscle training [4,5,6,7,8,9].

Miura et al. [10] presented a case who showed apparent amelioration of head drop after treatment sessions using a hybrid assistive limb (HAL; CYBERDYNE Inc., Tsukuba, Japan.). The HAL is a lower-limb exoskeleton type wearable robot that assists a patient in voluntary control of knee and hip joint motion. It has electrically driven power units at the bilateral hip and knee joints, which are actuated, amplifying the neuro-muscular activation of the relevant lower-limb muscles. HAL is known to be effective for gait improvement after neurological motion impairments including stroke [11,12], myelopathy in acute and chronic phases after spine surgery [13], spinal cord injury [14,15,16,17], and cerebral palsy [18]. However, regardless of these recent reports, improvement of DHS using HAL is reported only for a case so far [10]. In order to develop an effective conservative treatment for DHS, the purpose of this preliminary study is to apply HAL gait training of varied frequencies to DHS patients with slightly different radiograph examinations and to discuss plausibility of the treatment and possible responders and non-responders to the treatment.

## 2. Materials and Methods

### 2.1. Case Presentations

**Figure 1:**
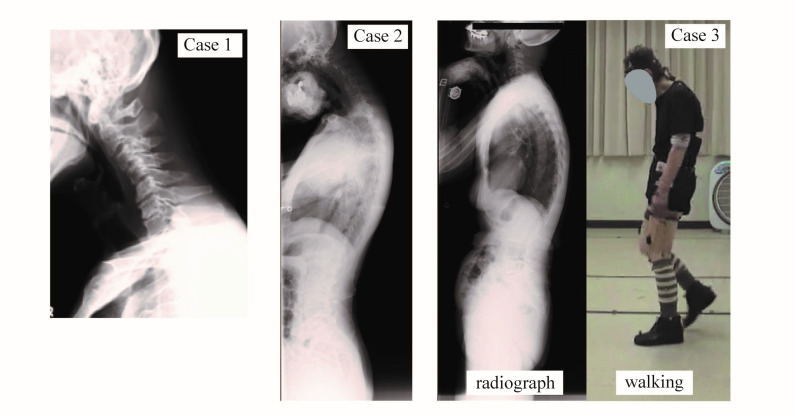
Radiograph images of the cervical spine showing dropped head syndromes of Case 1 (**left**), Case 2 (**middle**), and 3 (**right**). Case 3 managed to correct the cervical alignment momentarily when scanned (**left**, radiograph) but was not able to maintain the alignment and dropped head was observed during walking (**right**, photo).

Case 1: A 75-year-old man complained of dropped head and neck pain without neurological abnormalities. The patient’s activity of daily living was severely impaired by his dropped head. Physical therapy including cervical spine traction and training of neck extensor muscles was not effective. Cervical spine radiograph showed apparent kyphosis (Figure 1, Case 1). Magnetic resonance imaging (MRI) revealed no spinal cord compression.

Case 2: A 66-year-old man suffered from dropped head for one year. His dropped head was passively reducible with his hand. He had no traumatic history. Neurological examination did not reveal abnormalities at all. Conservative therapies such as wearing a collar and medication using non-steroidal anti-inflammatory drugs (NSAIDs) and an extract isolated from inflamed skin of rabbits inoculated with vaccinia virus (neurotropin) were not effective. A whole-spine radiograph in standing position revealed cervical kyphosis with mild degenerative change and lumbar hyperlordosis (Figure 1, Case 2). MRI showed no spinal canal stenosis.

Case 3: An 81-year-old woman presented dropped head for 7 years. Osteoporosis was diagnosed previously. Her dropped head particularly deteriorated in the evening. This was considered attributable to fatigue due to reduced endurance of extensor muscles. Conventional conservative therapy did not improve her symptom. The neurological examination was almost normal. A radiograph image in standing position obtained a relatively normal cervical alignment. Her dropped head was reducible for a moment while taking the radiograph (Figure 1, Case 3, left). However, it deteriorated by continuous walking (Figure 1, Case 3, right).

All three patients were Japanese inhabitants of Japanese origin categorized as Asians. Table 1. shows the patients demography, anthropometrics, and radiograph parameters at the time of inclusion.

### 2.2. HAL Gait Training

A double legged HAL was used in this study. HAL has an exoskeleton structure to be tightened on the pelvis, bilateral thighs, shanks and feet of the patient, with electric motors which assist sagittal motions of the hip and knee joints, weighing 14 kg in total. The electric motors are actuated by amplifying the bioelectric neuro-muscular activation of the relevant muscles to support voluntary joint motions. Disposable surface electrodes (Vitrode L, Nihon Kohden, Tokyo, Japan) were connected to the HAL’s cables and pasted bilaterally on the surface of hip flexor (tensor fasciae latae), hip extensor (gluteus maximus), knee extensor (vastus lateralis), and knee flexor (biceps femoris) muscles of the patient. The motor torques were generated in real-time: T_hip = G_hip_flex × A_hip_flex-G_hip_ext × A_hip_ext and T_knee = G_knee_flex × A_knee_flex-G_knee_ext × A_knee_ext are the hip and knee joint torques, where G* is the gain parameters, and A* is the filtered activation of the muscles, respectively. The gain parameters were manually adjusted for the patient’s comfort in each session.

**Figure 2:**
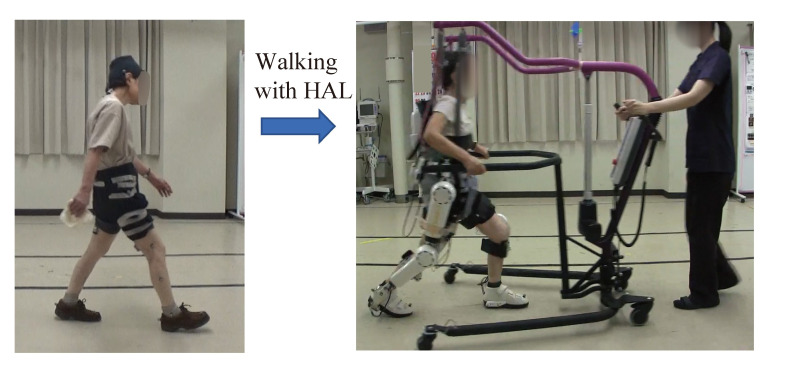
Gait training using HAL for patients with dropped head syndrome.

Ten sessions of gait training using HAL (Figure 2) was applied for each of the patients but with different frequencies (Table 1, ‘Frequency of HAL sessions’). Case 1 underwent two courses of HAL treatment; the 1st course was an intensive course of HAL treatment, with 5 sessions per week over two weeks. The 2nd course was at a relaxed frequency, with one session per month over 10 months. There was a 10 months interval between the 1st and the 2nd HAL courses. Case 2 underwent sessions at a moderate frequency; one session per week over 10 weeks. Case 3 underwent sessions at a rather relaxed frequency; one session per two to three weeks over 6 months.

One session lasted about one hour, including 10 m walking test without using HAL, attachment of HAL, gait training using HAL, detachment of HAL, and 10 m walking test without using HAL. HAL gait training included continuous over-ground walking at a comfortable pace on a 25 m oval course with rest interval. During HAL gait training, an All-in-One Walking Trainer (Ropox A/S, Naestved, Denmark) was used with a harness to prevent falls. Weight support was not provided during gait training. An assistant pulled the All-in-One Walking Trainer in accordance with the patient’s gait speed. The HAL and the All-in-One devices are independent systems without any systematically shared control between them.

### 2.3. Assessment of Cervical Alignment during Gait

Assessment of cervical alignment during gait was two-fold in this study. Immediate effect of HAL training on cervical alignment in each single session was evaluated by comparing cervical alignment during a 10 m walking test just before (HAL_PRE) and after (HAL_POST) HAL gait training in each session. Lasting effect after the HAL intervention period was evaluated by comparing cervical alignment during gait before the first session (PRE) and after the last session (POST). Gait of PRE and POST was assessed by a long-time walking protocol for evaluation of postural balance during gait [19,20,21], where the patient was asked to keep walking as long as possible at a comfortable pace on a flat ground surface repeating 10 m straight walk and semi-circular turning until perception of fatigue or pain. The long-time walking protocol was adopted, because the spinal alignment during gait is considered more directly related to ADLs than that in static standing posture [19,20,21].

### 2.4. Measurement and Data Processing

**Figure 3:**
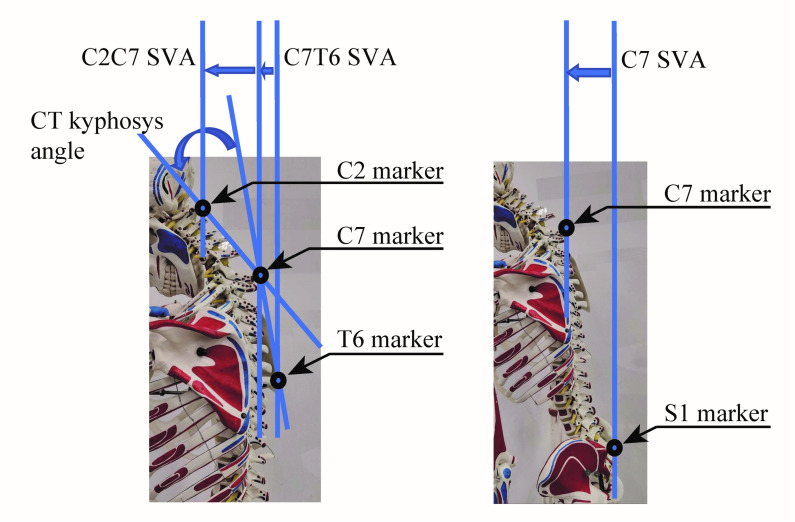
Definition of spinal parameters extracted from the motion capture data for calculation of cervical alignment during walking. During walking, optical markers of motion capture were attached to the surface of the C2, C7, T6, and S1 spinous processes. The measured marker positions were used to calculate C2C7SVA, CT kyphosis angle, C7T6SVA, and C7SVA.

Cervical alignment during gait was recorded using a motion capture system (Vicon MX with 16 T20s cameras, Vicon, Oxford, UK) sampling at 100 Hz. Auto-reflective markers (14 mm diameter) were placed on the cervical spine; C2, C7, and T6 spinous processes in HAL_PRE and HAL_POST, C2, C7, T6, and S1 spinous processes in PRE and POST (Figure 3) and on the feet; lateral malleolus for the ankle, posterior peak of the calcaneus for the heel, and the second metatarsal bone of the toe. The bone landmarks were detected by palpation. The cervical markers were pasted on the skin surface using a double-sided scotch (3M health care, 3M, Maplewood, USA). The thoracic and pelvic markers were pasted on a tight-fitting shirt and short pants respectively. A single side scotch (Yukiban, Nitto, Tokyo, Japan) was used to apply tension on the clothes when necessary to prevent loosening of them around the markers. Marker labeling was performed using Vicon Nexus software (version 2.2.3). The rest of the processing was performed using custom scripts on MATLAB 8.4 R2014b (Mathworks, Natick, MA, USA).

Spinal parameters of cervical SVA (sagittal vertical axis) (C2C7SVA), upper thoracic SVA (C7T6 SVA), spinal SVA (C7SVA), and cervico-thoracic kyphosis angle (CT kyphosis) during walking were extracted from the motion capture data. The SVA parameters were calculated respectively as the sagittal distance between the C2 and C7 markers, C7 and T6 markers, and C7 and S1 markers (Figure 3). CT kyphosis was calculated as the angle between the line that goes through C2 and C7 and the line that goes through C7 and T6, within the sagittal plane. Gait cycles were detected, according to the foot markers, as starting from a left heel landing and ending with the next left heel landing. The spinal parameters were segmented into gait cycles and averaged within the cycles to obtain representative alignment within a cycle. Then, the value is averaged among the extracted gait cycles to obtain SVA parameters of a gait.

For the long-time walking data of the PRE and POST assessments, the long data tracks were segmented first into each straight 10 m walking before labeling the markers. The SVA parameters were obtained using the above procedure for each of the 10 m walking laps.

### 2.5. Statistical Analyses

A paired *t*-test was used to compare the cervical parameters of immediately before and after using HAL in each session (HAL_PRE and HAL_POST). To compare the cervical parameters during long-time walking between the PRE and POST assessments, a multiple regression was first used. When the multiple regression does not show significant interaction between the walking distance (lap) and the group condition (PRE and POST), then the dependence of the SVA parameters on lap and PRE-POST were independently examined using the regression coefficients. When interaction was found, post-hoc analysis was performed using a simple unpaired *t*-test. *p*-values smaller than 0.05 were considered as detection of statistically significant difference. *p*-values smaller than 0.1 and greater than 0.05 were considered as tendency of difference. Statistical processing was performed using custom scripts on R version 3.6.0.

## 3. Results

### 3.1. Immediate Effects

**Figure 4:**
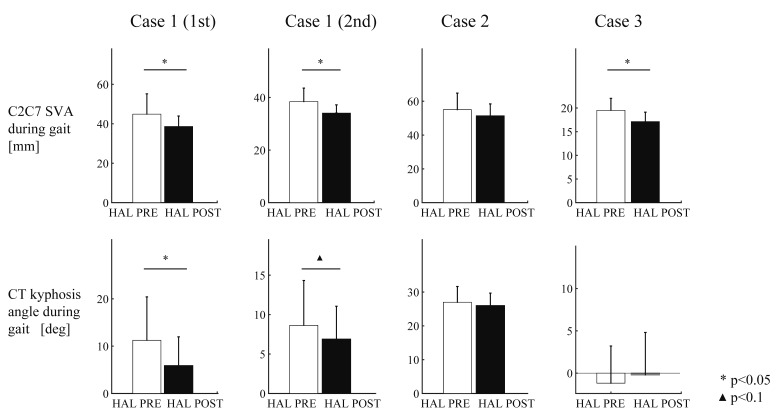
Immediate effects of HAL on cervical alignment during gait. C2C7SVA and CT kyphosis angle were compared between 10 m walking tests of just before and after HAL gait training in each of 10 HAL sessions.

Immediate reduction of C2C7SVA in single HAL sessions (Figure 4) was observed with statistical significance in Case 1 (1st) (mean values: HAL_PRE 45 mm and HAL_POST 39 mm, *p* = 0.022), Case 1 (2nd) (38 and 34 mm, *p* = 0.007), and Case 3 (19 and 17 mm, *p* = 0.001). Reduction of C2C7SVA was observed in Case 2 but without statistical significance (55 and 51 mm, *p* = 0.271). C7T6SVA increased in Case 1(1st) (97 and 143 mm, *p* = 0.00004) and decreased in Case 1 (2nd) (99 and 94 mm, *p* = 0.045) and in Case 2 (44 and 37 mm, *p* = 0.047). CT kyphosis angle decreased in Case 1 (1st) (11.2 and 5.9 deg, *p* = 0.0048) and tended to decrease in Case 1 (2nd) (8.6 and 6.8 deg, *p* = 0.091).

### 3.2. Lasting Effects

**Figure 5:**
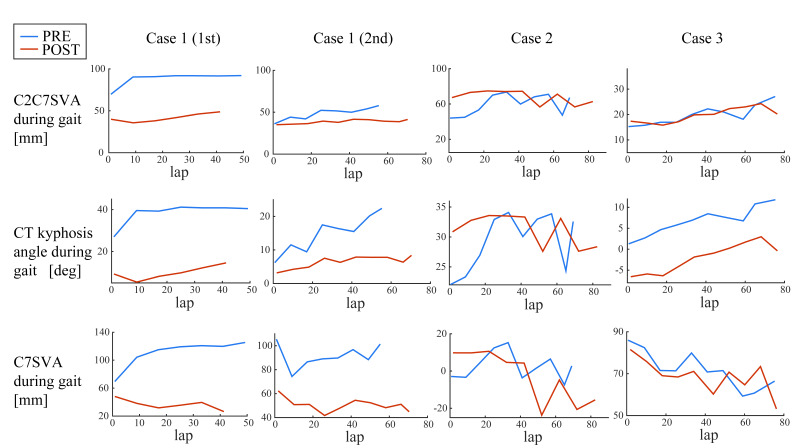
Lasting effects of HAL on cervical alignment during gait. C2C7SVA, CT kyphosis angle, and C7SVA during long-distance walking were compared between before the first and after the last HAL gait training sessions.

Case 1 (1st) showed reduction of the spinal parameters during long-time walking in POST compared with PRE; C2C7SVA (Figure 5, regression coefficients: PRE 81 mm and POST 36 mm, *p* = 1.57 × 10^−5^), CT kyphosis angle (regression coef. 34 and 6.2 deg, *p* = 1.97 × 10^−5^), and C7SVA (mean values 111 and 37 mm, *p* = 5.60 × 10^−8^). Case 1 (2nd) also showed reduction of C2C7SVA (mean values 49 and 37 mm, *p* = 0.0024), CT kyphosis angle (mean values 15 and 6.4 deg, *p* = 0.0013), and C7SVA (regression coef. 88 and 54 mm, *p* = 0.000237). Case 2 did not show statistically significant reduction. Case 3 showed reduction of CT kyphosis angle (regression coef. 2.1 and −7.0 deg, *p* = 4.11 × 10^−7^) but not of C2C7SVA or C7SVA. The *p*-values of the statistical tests are summarized in Table 2.

## 4. Discussion

Although there are multiple recent reports on functional gait recovery after using HAL in patients with mobility issues due to various neurological disorders [11,12,13,14,15,16,17,18], there are only very few studies focusing on using HAL for spinal deformity. Evaluating immediate and lasting effects of gait treatment using HAL for spinal deformity, this study investigated the cervical alignment during gait of three dropped head syndrome (DHS) patients who underwent a total of 10 HAL gait sessions at different frequencies. Case 1 underwent HAL training at the frequency of 5 times per week in the first application of HAL, and once per month in the second application of HAL, 10 months after the first application of HAL. Case 2 underwent HAL sessions once per week and Case 3 once per 2.5 weeks. The immediate effect was defined as the effect of HAL observed in each single session, while the lasting effect was defined as the effect of HAL after a course of treatment.

Lasting effects of HAL for DHS evaluated by comparing cervical alignment during PRE and POST long-time walking tests showed positive effect of HAL on the reduction of C2C7SVA, CT (cervico-thoracic) kyphosis, and C7SVA in Case 1 (1st) and Case 1 (2nd). Geometrically, C2C7SVA is determined by the CT kyphosis and the thoracic alignment below C7 marker. From this viewpoint, the reduction of C2C7SVA in this case may have been achieved by the two factors; reduction of CT kyphosis and the improved control of the trunk posture which is indicated by the reduction of C7SVA. The results showed reduced C7SVA and CT kyphosis, indicating straightened trunk posture and extended neck resulting in the reduced C2C7SVA. In this sense, improvement of cervico- thoracic alignment during gait was observed in Case 1.

**Figure 6:**
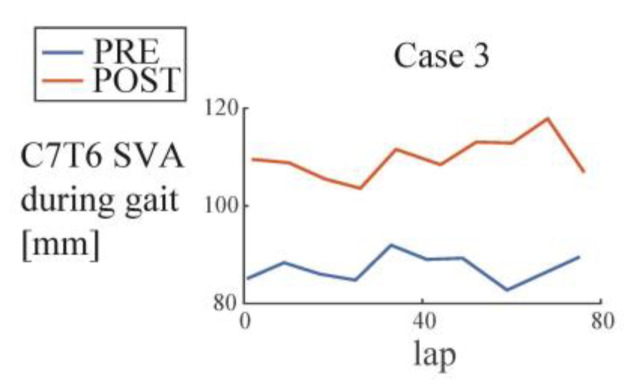
Lasting effects of HAL on thoracic alignment during gait (Case 3, C7T6SVA).

Case 3 showed decreased CT kyphosis, however, C2C7SVA did not decrease. C7SVA did not show decrease or increase, but a closer look at the C7T6SVA (upper thoracic SVA) showed significant increase (Figure 6, regression coef. PRE 87 mm and POST 107 mm, *p* = 4.67 × 10^−6^), suggesting increased kyphosis at the thoracic level. It suggests that improvement of CT kyphosis is necessary but is insufficient for the improvement of C2C7SVA, because the worsened thoracic kyphosis can cancel out the effect of improved CT kyphosis. It suggests that it needs to be accompanied by improved or at least unchanged thoracic kyphosis to obtain improvement of C2C7SVA.

Case 2 did not show statistically significant change of cervical alignment in the PRE–POST comparison. Radiograph assessment in static standing posture (Table 1) showed the patient’s shallow T1 slope and posteriorly inclined posture of the trunk with lumbar hyperlordosis. We considered that in this posture, the trunk was already tilting to the possible posterior extreme to compensate the dropped head, and because of this, it did not have room for the trunk posture to serve as a compensatory adjuster to support cervical alignment during gait.

Positive immediate effects on the reduction of C2C7SVA was observed in Case 1 (1st), Case 1 (2nd), and Case 3, and on the reduction of CT kyphosis angle in Case 1 (1st), Case 1 (2nd). The apparent immediate effects of HAL suggest that the effect of walking with HAL can cause some changes in the coordination of neck and leg muscles [22] during gait, rather than training of the neck extensor muscle strength. Immediate effect of HAL is reported also on the improvement of gait [18] and upper limb motion [23] in children with cerebral palsy whose problems are in the coordinated control of the limb muscles rather than the muscle force.

The relationship between the immediate and lasting effects suggests that immediate improvement of C2C7SVA and CT kyphosis in each HAL session may be related to the improvement of C2C7SVA during walking in POST evaluation. This relationship can be used for detection of responders, providing a reference indicator about if further continued prescription of HAL training is meaningful for each patient in the middle of a course of HAL training sessions. This also may provide a helpful information for diagnosing types of DHS.

On the other hand, even though the participants of this study underwent HAL treatments at different frequencies, the relationship between the frequency and outcomes of each participant was not clear. Hence, the optimal frequency of HAL for DHS remains as an open question. A recent study on spinal cord injury patients [14] reported that combination of an intensive frequency (once per day) at the beginning phase for 12 weeks and a mild frequency (once per week) at the continuing phase can be a candidate of optimal schedule, to obtain improvement in the intensive phase and maintain the improved gait through the mild phase. Exploration of such planning for DHS patients remains for future investigation.

Another aspect for consideration is multidimensional assessment of subjective perception of the treatment by the patients. A review article on nonspecific chronic low back pain [24] stresses the importance of assessing biological, functional, psychosocial, and social outcomes including disability and performance of ADL, muscular strength, adiposity, kinesiophobia, pain catastrophizing, self-efficacy, depression, sleep quality, social function, work absenteeism, and quality of life, in addition to the conventionally assessed pain intensity. In the case of DHS, the chronic syndrome also affects ADL and QOL and therefore psychological and social aspects. Evaluation of these aspects in addition to cervical alignment would be informative in assessing treatment outcomes for DHS.

A limitation of the study is that, as a preliminary report of only three cases, the results of the study are not reasonably generalizable. However, despite the significant affects brought by the pathology to patients’ ADL, reports on DHS treatment are mostly case report or case series report, due to the limited frequency of DHS occurrence in general. This is suggested by a recent systematic review [2] where the description of a total of 129 patients was found among 74 studies, indicating that the average number of patients per study is less than two. In this regard, accumulation of reports from abundant sites would be informative even though the number of patients per study is limited, to serve as a basis for future studies with larger number of patients. Another limitation is that biomechanics behind the changes and differences was not evaluated in this study. Measurement of trunk muscle activities related to spinal alignment during gait for analysis of kinetics would be able to provide further information on the mechanism of the treatment, which is left for future research.

## 5. Conclusions

The results of the preliminary study suggested that gait training using HAL may be an effective option of conservative treatment for a part of DHS patients. They also suggested, on the other side, that a lack of immediate effects on the cervical alignment and a lack of ability to perform compensatory trunk motion may indicate a non-responding patient.

## Figures and Tables

**Table 1:** Case descriptions: age, sex, height, weight, radiograph assessment of cervical spine at the time of inclusion, and frequency of Hybrid Assistive Limb (HAL) sessions.

Case	Case 1	Case 2	Case 3
Age (y)	75	66	81
Sex	M	M	F
Height (cm)	169	162	145
Weight (kg)	60	49	37
Head-C7 SVA (mm)	+115	+117	+8.2
Cervical Kyphosis	40°	37°	0.3°
T1 slope	39°	0°	11°
C7 SVA (mm)	−21	−64	4.9
Lumbar Lordosis	40°	63°	28°
Number of HAL sessions	10	10	10
Frequency of HAL sessions	(1st) 5/week (2nd) 1/month	1/week	1/2–3 weeks
Duration of HAL sessions	(1st) 2 weeks (2nd) 10 months	10 weeks	6 months

**Table 2:** *p*-values from a multiple regression analysis of cervical alignment during gait in PRE (before the first HAL session) and POST (after the last HAL session) evaluations, corresponding to Figure 5 and Figure 6.

Case	Outcome	Predictor			Post-Hoc
		(Interaction)	PRE-POST	Lap	PRE-POST
Case 1 (1st)	C2C7 SVA	0.9081	**1.57 × 10^−5^**	**0.0347**	n/a
	CT kyphosis	0.8772	**1.97 × 10^−5^**	**0.0331**	n/a
	C7 SVA	**0.00789**	n/a	n/a	**5.60 × 10^−8^**
Case 1 (2nd)	C2C7 SVA	**0.000403**	n/a	n/a	**0.0024**
	CT kyphosis	**0.000247**	n/a	n/a	**0.0013**
	C7 SVA	0.297684	**0.000237**	0.490349	n/a
Case 2	C2C7 SVA	**0.0453**	n/a	n/a	0.9523
	CT kyphosis	**0.0339**	n/a	n/a	0.8481
	C7 SVA	**0.0184**	n/a	n/a	0.1486
Case 3	C2C7 SVA	0.299	0.448	**5.68 × 10^−5^**	n/a
	CT kyphosis	0.9250	**4.11 × 10^−7^**	**2.72 × 10^−6^**	n/a
	C7 SVA	0.45344	0.23803	**0.00127**	n/a
	C7T6 SVA	0.305	**4.67 × 10^−6^**	0.886	n/a

Bold letters indicate *p*-values less than 0.05.
